# A metagenomics-based workflow for the detection and genomic characterization of GBS in raw freshwater fish

**DOI:** 10.1128/spectrum.03276-23

**Published:** 2024-05-07

**Authors:** Kae Hwan Sim, Jiaying Ho, Jia Qi Lim, Sheot Harn Chan, Angela Li, Kern Rei Chng

**Affiliations:** 1 National Centre for Food Science, Singapore Food Agency, Singapore, Singapore; 2 Department of Food Science & Technology, Faculty of Science, National University of Singapore, Singapore, Singapore; 3 Department of Biological Sciences, Faculty of Science, National University of Singapore, Singapore, Singapore; The Pennsylvania State University, University Park, Pennsylvania, USA

**Keywords:** metagenomics, foodborne pathogen, group B *Streptococcus*, biosurveillance, food safety

## Abstract

The unexpected foodborne outbreak in Singapore in 2015 has accentuated Group B *Streptococcus* (GBS, *Streptococcus agalactiae*) sequence type 283 as an emerging foodborne pathogen transmitted via the consumption of contaminated raw freshwater fish. Isolation-based workflows utilizing conventional microbiological and whole-genome sequencing methods are commonly used to support biosurveillance efforts critical for the control management of this emerging foodborne pathogen. However, these isolation-based workflows tend to have relatively long turnaround times that hamper a timely response for implementing risk mitigation. To address this gap, we have developed a metagenomics-based workflow for the simultaneous detection and genomic characterization of GBS in raw freshwater fish. Notably, our validation results showed that this metagenomics-based workflow could achieve comparable accuracy and potentially better detection limits while halving the turnaround time (from 2 weeks to 5 days) relative to an isolation-based workflow. The metagenomics-based workflow was also successfully adapted for use on a portable long-read nanopore sequencer, demonstrating its potential applicability for real-time point-of-need testing. Using GBS in freshwater fish as an example, this work represents a proof-of-concept study that supports the feasibility and validity of metagenomics as a rapid and accurate test methodology for the detection and genomic characterization of foodborne pathogens in complex food matrices.

**IMPORTANCE:**

The need for a rapid and accurate food microbiological testing method is apparent for a timely and effective foodborne outbreak response. This is particularly relevant for emerging foodborne pathogens such as Group B *Streptococcus* (GBS) whose associated food safety risk might be undercharacterized. By using GBS in raw freshwater fish as a case example, this study describes the development of a metagenomics-based workflow for rapid food microbiological safety testing and surveillance. This study can inform as a working model for various foodborne pathogens in other complex food matrices, paving the way for future methodological development of metagenomics for food microbiological safety testing.

## INTRODUCTION

The first reported foodborne outbreak of invasive Group B *Streptococcus* (GBS, *Streptococcus agalactiae*) disease occurred in Singapore in 2015 (1). The outbreak affected more than 160 people and was directly linked to the consumption of ready-to-eat raw freshwater fish contaminated with GBS sequence type (ST) 283 (ST283) (2, 3). The scale and severity of this unexpected foodborne outbreak brought attention to GBS ST283 as an emerging foodborne pathogen that had previously been overlooked despite its widespread presence across Southeast Asia as a significant pathogen causing disease in humans and freshwater fish (4). GBS ST283 places a significant burden on both public health and the economy as infection in healthy humans can result in serious illnesses such as meningitis while infection in fish can cause high mortalities in aquaculture (4). As an emerging foodborne pathogen that is currently understudied, a multitude of known data gaps can confound the food safety risks associated with GBS ST283 (5). Biosurveillance and risk analysis programs represent a key approach to gaining situational awareness, addressing uncertainties, and directing pre-emptive actions for the management of food safety risks posed by GBS ST283 (2). Surveillance data are highly time-sensitive as a rapid response is critical to enable timely public health decision-making to decisively safeguard public health (6).

Conventional isolation-based microbiological testing methods, which require a long turnaround time (TAT), are currently still the mainstay for the detection and characterization of GBS in food (7). These methods require laborious culturing, enrichment, and isolation steps that are time-consuming and have low throughput (8). In contrast, metagenomics-based methods enable the simultaneous detection and characterization of the entire microbial community (9) and, therefore, offer immense potential to scale biosurveillance efforts (10). While large-scale applications of metagenomics for clinical diagnostics (11) and environmental microbiome surveillance (12) are not uncommon, the typical low abundance of foodborne pathogens in food microbiomes posed serious technical challenges to the development of metagenomics-based methods for food microbiological testing (13). To circumvent this, there have been ongoing efforts to advance the development of metagenomics-based analytics on enriched food culture fractions (i.e., quasimetagenomics) (14–17). However, owing to the complexity of food matrices and the food microbiomes, the culturing strategy (e.g., media composition and growth conditions) for quasimetagenomics has to be optimized according to the specific food matrices and target foodborne pathogens (15, 18). Despite the known difficulties in developing such assays, the potential benefit of applying metagenomics to reduce response time to support foodborne outbreak investigation and biosurveillance has been well demonstrated (16).

In this work, we have developed a metagenomics-based workflow for the rapid detection and genomic characterization of GBS in raw freshwater fish. The performance of the metagenomics-based workflow was benchmarked against an isolation-based workflow through artificial GBS spiked-in studies and further validated with real-world freshwater fish samples. The results showed that we were able to optimize the GBS enrichment process to a single culturing step for metagenomics-based detection and characterization of GBS in raw freshwater fish. Coupled with our proposed bioinformatics analytical strategy, the developed metagenomics-based workflow was able to substantially shorten the TAT, while achieving limits of detection (LOD) and accuracy comparable to, if not better than, the isolation-based workflow. To demonstrate the feasibility of the metagenomics-based workflow for rapid point-of-need food testing, we also conducted proof-of-concept work to adapt the workflow to a long-read portable sequencer that can be used for real-time on-site applications.

## MATERIALS AND METHODS

### Isolation-based workflow for GBS in freshwater fish

Raw freshwater fish samples were sampled from the wet markets and supermarkets in Singapore between April and December 2022. All fish samples were thawed on ice before sample processing and subsequently cultured for GBS, following a modified published protocol (7). Briefly, 25 g of fish skin and meat was aseptically cut from the fish samples and homogenized in 225 mL of Todd Hewitt broth (THB) supplemented with 7.5 µg/mL of colistin sulfate and 5 µg/mL of nalidixic acid [modified colistin sulfate and nalidixic acid (CNA) selective supplement; Oxoid, Basingstoke, United Kingdom] using a stomacher (model 400; Seward Medical, Worthing, United Kingdom) at 230 rpm for 30 sec. The samples were incubated at 37°C for 20 ± 2 h without shaking. After primary enrichment, 1 mL of enriched culture was mixed with 5 mL of brain heart infusion (BHI) broth and incubated at 37°C for another 20 ± 2 h without shaking. The enriched culture was diluted 10,000 times in Butterfield’s Buffer (pH 7.2); 1 mL of the 10^−4^ dilution was plated on ChromID Strepto B selective agar (bioMérieux, Marcy l’Etoile, France), followed by incubation at 37°C for 24–48 h. The suspected GBS isolates were purified and primarily tested using a Streptococcal Grouping Kit (Oxoid, Basingstoke, United Kingdom). If the suspected isolates were classified as Lancefield group B, they were harvested and stored in 30% glycerol stock at −80°C for downstream molecular identification of GBS and ST characterization. The DNA samples of suspected GBS isolates were extracted using the commercial kit DNeasy PowerSoil Pro (Qiagen, Hilden, Germany) according to the manufacturer’s protocol. The taxonomic identity of suspected isolates was confirmed using Sanger sequencing. Bacterial 16S rRNA gene sequences were amplified using the primer pair 338F (5′-ACTYCTACGGRAGGCWGC-3′) and 1061R (5′-CRRCACGAGCTGACGAC-3′) based on a previously published report (17). The consensus fasta sequence was deduced and searched against (blastn) the NCBI 16S rRNA sequence database to verify the species identity. Next, multi-locus sequence typing (MLST) was performed to confirm the ST of GBS-positive isolates. Seven housekeeping genes (*adhP*, *pheS*, *atr*, *glnA*, *sdhA*, *glcK,* and *tkt*) were amplified and sent for Sanger sequencing as described previously (19). The consensus fasta sequences for the seven MLST genes were searched against (blastn) the PubMLST *Streptococcus agalactiae* database to characterize the ST of the GBS isolates (20).

### Sample preparation for GBS spike-in experiments

The GBS spike-in experiments were conducted using a single GBS ST283 strain previously isolated from a freshwater fish sample. Briefly, GBS was streaked on a tryptone soy agar (TSA) plate and incubated at 37°C overnight. One colony was aseptically picked and subsequently inoculated and incubated in 20 mL of THB (with modified CNA selective supplement) at 37°C with 150 rpm constant shaking to obtain an optical density at 600 nm (OD_600 nm_) of 0.8–1.0. The broth was then diluted to OD_600 nm_ of 0.28 (approximately 10^8^ CFU/mL). An enumeration of 100 µL of the dilution 10^−6^ was performed in duplicate on a TSA plate incubated at 37°C for 18 ± 2 h to quantitate spike amounts. A total of 125 g of fish skin and meat was sampled and evenly distributed into five stomacher bags: one portion was not artificially contaminated and served as the “non-spike” control while the four other portions were spiked with varying GBS amounts of 10^1^–10^2^ CFU, 10^2^–10^3^ CFU, 10^3^–10^4^ CFU, and 10^4^–10^5^ CFU, respectively.

### DNA extraction

The commercial kit DNeasy PowerSoil Pro (Qiagen, Hilden, Germany) was used for DNA extraction according to the manufacturer’s protocol. The DNA purity and concentration were measured on a NanoDrop 2000c spectrophotometer (Thermo Fisher Scientific Ltd., Paisley, United Kingdom) and Qubit 1.0 (Thermo Fisher Scientific Ltd., Paisley, United Kingdom) using the Qubit dsDNA HS Assay Kit (Thermo Fisher Scientific Ltd., Paisley, United Kingdom).

### Metagenome sequencing

For Illumina shotgun sequencing, the whole metagenome library preparation and sequencing were performed by NovogeneAIT Genomics Singapore Pte Ltd. The assessment of microbial DNA concentration, purity, and degradation was performed using Qubit 2.0 (Thermo Fisher Scientific, United States), NanoDrop (Thermo Fisher Scientific, United States), and gel electrophoresis. The library was prepared using the TruSeq DNA PCR-Free Prep Kit (Illumina, United States) and checked using Qubit and real-time PCR for quantification and bioanalyzer for size distribution detection. The prepared paired-end sequencing library was sequenced on the NovaSeq 6000 platform (2 × 150 bp chemistry) (Illumina, United States) to generate approximately 6 Gbases per sample.

For nanopore long-read sequencing, DNA libraries were prepared in-house according to the ligation sequencing gDNA–native barcoding (SQK-LSK109 with EXP-NBD104 and EXP-NBD114, version NBE_9065_v109_revAM_14Aug2019, updated on 4 July 2022) protocol from Oxford Nanopore Technologies (Oxford Nanopore Technologies Ltd., Oxford, United Kingdom), and the sequencing run was performed on a MinION Mk1c (Oxford Nanopore Technologies Ltd., Oxford, United Kingdom) for approximately 30 h.

### Analysis of DNA sequencing data

The Illumina shotgun sequencing reads were put through an in-house analysis workflow. Briefly, raw fastq files were processed using fastqc (version 0.11.9) (21) and fastp (version 0.23.2; -q 30 --detect_adapter_for_pe --cut_right) (22) to remove sequencing adapters and low-quality (phred score <30) reads. The preprocessed high-quality reads were subjected to taxonomic profiling for the identification of GBS in samples using MetaPhlAn (version 4.0.2) with the mpa_vJan21_CHOCOPhlAnSGB_202103 database (downloaded on 28 November 2022) (23), where hits with relative abundance of <0.1% were considered absent. The preprocessed reads were subsequently mapped to the reference GBS genomes (Table S1, downloaded from NCBI on 17 October 2022) using bwa (version 0.7.17; default parameters) (24). Mapped reads were extracted using samclip (version 0.4.0) (25) and samtools (version 1.15.1) (26) with default settings.

For nanopore long-read sequencing, the raw fast5 reads were base-called and demultiplexed using Guppy (version 5.0.15+a642d3b) with dna_r9.4.1_450bps_sup super high accuracy model, which would improve the raw read accuracy but is computationally expensive, to produce fastq files for each sample (27). Reads were classified as pass or fail based on a minimum quality score of 10. The fastq files containing passed reads with more than 1,000 bp per read were merged into one per sample. The preprocessed fastq reads were taxonomically classified to identify GBS in samples using Kraken2 (version 2.0.9beta) with the MiniKraken_v2 database (updated on April 2019, downloaded on 29 April 2022) (28). The preprocessed fastq reads were subsequently mapped to the reference GBS genomes using minimap2 (version 2.24; default parameters) (29). Mapped reads were extracted using CoverM (version 0.6.1) (30) and samtools (version 1.16.1) (31) with default settings.

### Genome assembly, taxonomic identification, and sequence typing analyses

Illumina shotgun sequencing reads mapped to the reference GBS genomes were assembled using SPAdes (version 3.15.4; default parameters) (32). For nanopore long-read sequencing, reads mapped to the reference GBS genomes were assembled using flye (version 2.9; --nano_hq -g 2.1m) (33), and the assembled contigs were polished using medaka (version 1.4.4; -m r941_min_sup_g507) (34). Taxonomic identification of the assembled genomes was performed using GTDB-Tk (version 2.0.0) with the Genome Taxonomy database (version R207, downloaded on 7 April 2022) (35).

For assembly-based metagenomics analysis of Illumina shotgun sequencing reads, genome assembly was conducted using metaSPAdes (version 3.15.4; default parameters) (32). The assembled contigs were binned using metaWRAP (version 1.3; default parameters) (36). Taxonomic identification of bins was conducted using GTDB-Tk (version 2.0.0) with the Genome Taxonomy database (version R207) (35).

The quality of the genome assemblies was checked using QUAST (version 5.0.2) (37) and CheckM (version 1.1.9; completeness >90% and contamination <5%) (38). Sequence typing of GBS was carried out using mlst (version 2.22.0; default parameters) (20, 39).

### SNP analysis for GBS strain resolution

Samples with at least 20× GBS coverage were subjected to single nucleotide polymorphism (SNP) analysis. SNPs were identified on the basis of mapping the extracted GBS (Illumina) reads using bwa (version 0.7.17; soft-clipped reads, and reads with three or more mismatches were filtered out) to a reference GBS genome of a different ST and variant calling using bcftools (version 1.17; minimum read depth of 10) (40).

### Diversity analysis of raw freshwater fish culture microbiomes

Alpha (Shannon and Simpson indexes) and beta (Bray–Curtis dissimilarity) diversities were calculated using the diversity() function in “vegan” R package (41).

### Statistical analysis

Analysis was performed using R unless otherwise specified.

## RESULTS

### A metagenomics-based workflow for detection and characterization of GBS in raw freshwater fish

**Fig 1:**
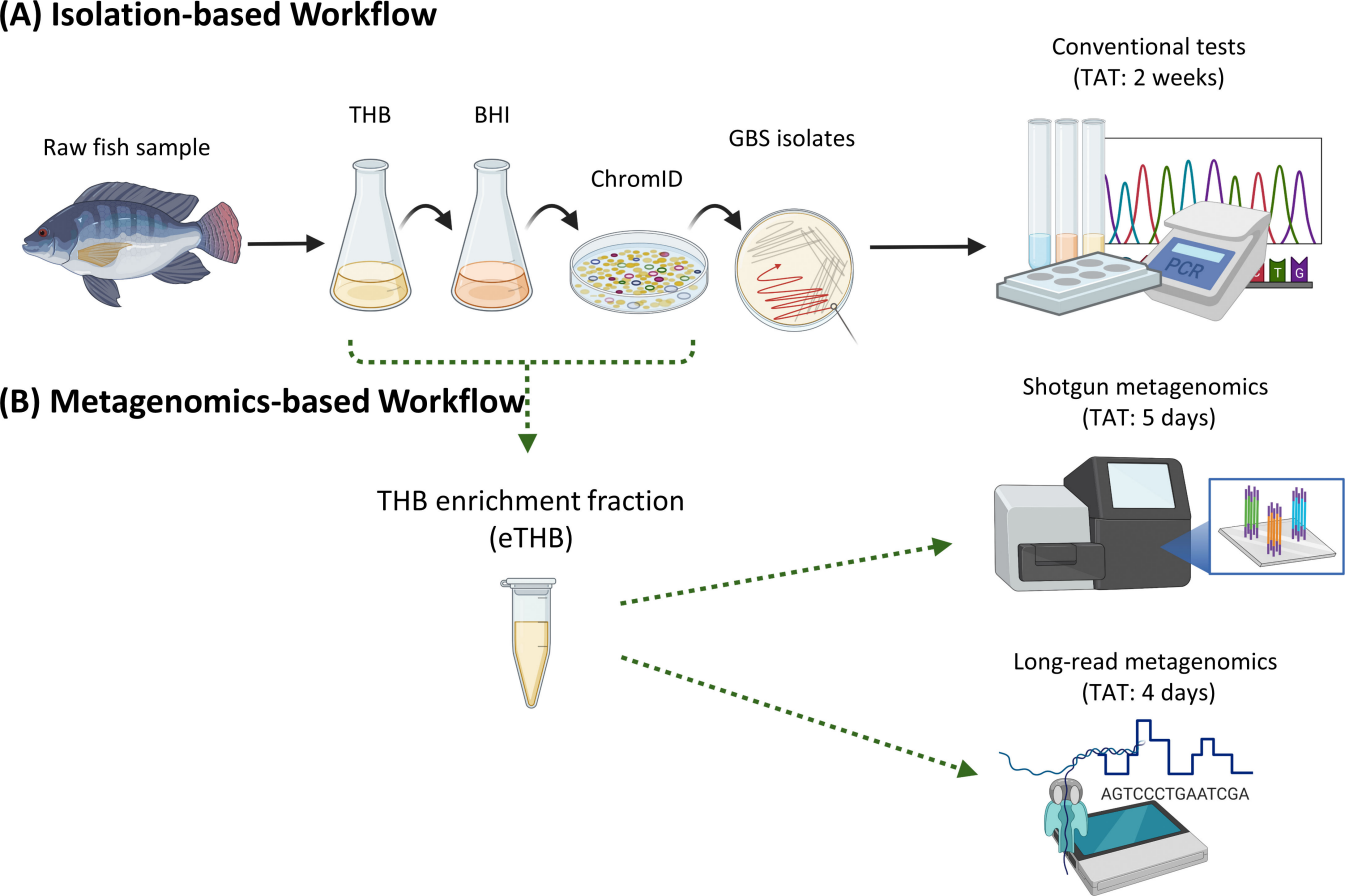
Graphical illustration showing (**A**) an isolation-based workflow and (**B**) a proposed metagenomics-based workflow for the detection and characterization of *Streptococcus agalactiae* (GBS) in raw freshwater fish. The metagenomics-based workflow offers a substantially shorter turnaround time when compared to the isolation-based workflow. The figure is created with BioRender.

The isolation of GBS from raw freshwater fish is constrained by the presence of diverse microbial species and the low abundance of GBS in its microbiome (42). Consequently, a conventional isolation-based workflow would incorporate several consecutive selective enrichment cultures for growing GBS, enabling isolation (Fig. 1A). The requirement for multiple culturing steps inadvertently results in a long TAT for GBS isolation (Fig. 1A). As the low abundance of GBS in fish microbiome would also limit the detection of GBS by direct metagenomics, we rationalized for a quasimetagenomics approach to develop the metagenomics-based workflow (Fig. 1B). Accordingly, the culturing enrichment strategy would be critical to the LOD and TAT of the metagenomics-based workflow.

**Fig 2:**
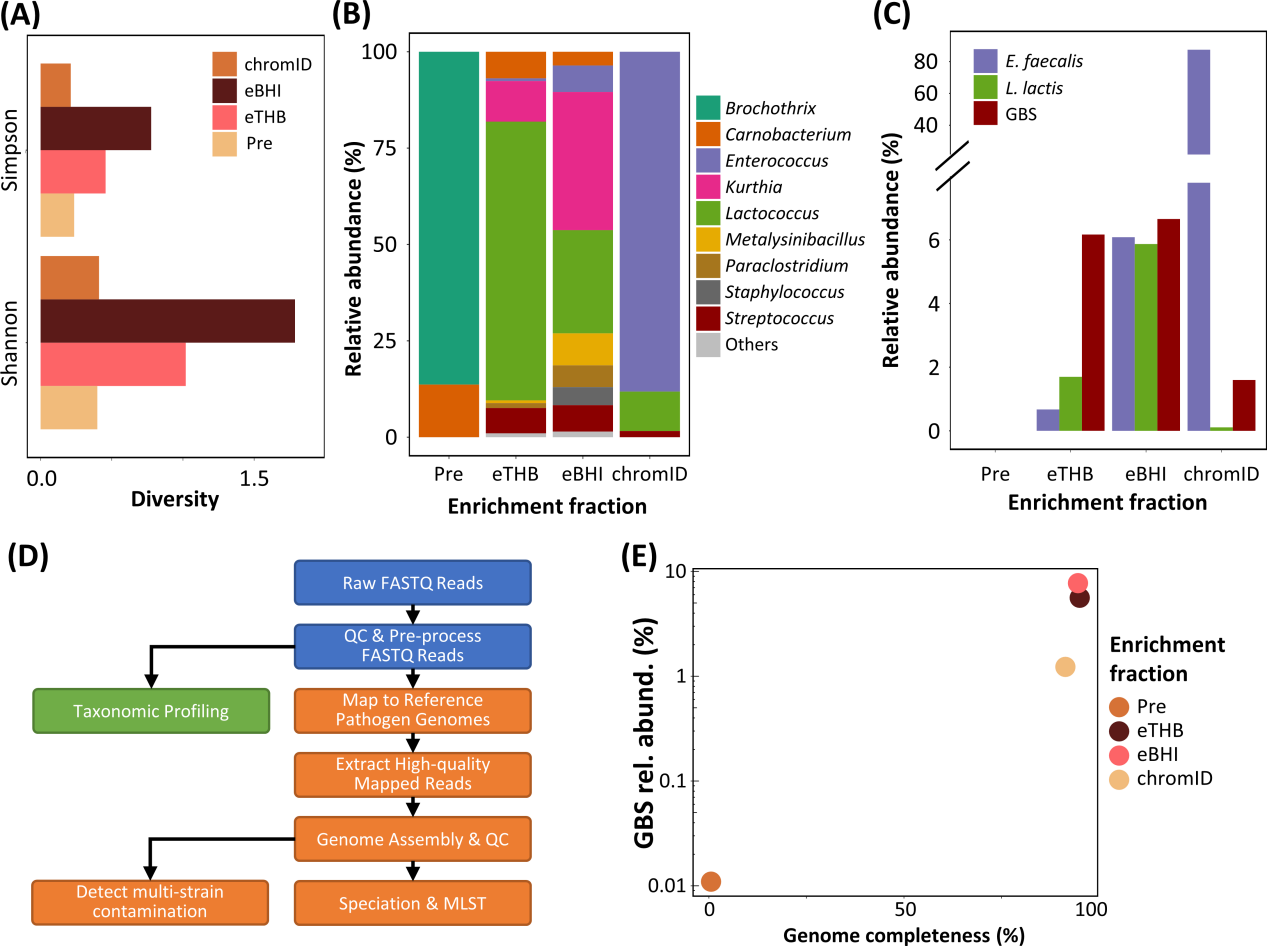
Characterization of the metagenomics-based workflow for insights to optimize detection diversity coverage, limit of detection, accuracy, and turnaround time of targeted microbes in raw freshwater fish samples naturally contaminated with GBS. (**A**) Alpha diversity analysis of the four different enrichment culture fractions. (**B**) Taxonomic profiles of the four different enrichment culture fractions at the genus level. (**C**) Relative abundances of targeted species (*E. faecalis*, *L. lactis*, and GBS) in the four different enrichment culture fractions. (**D**) Outline of the (default; mapping-based) metagenomics-based bioinformatics analytics. (**E**) Metagenomics-based workflow output of the relative abundances and completeness of recovered genomes for GBS in the four different enrichment culture fractions. Pre, pre-enrichment fraction; eTHB, THB enrichment culture fraction; eBHI, BHI enrichment culture fraction; chromID, chromogenic Strepto B agar culture fraction.

As a pilot, we retrospectively characterized, via metagenomics analysis, the different enrichment culture fractions [obtained from an isolation-based workflow (1)] of a raw freshwater fish sample naturally contaminated with GBS in order to identify a suitable enrichment culture fraction for quasimetagenomics (Fig. 1B). We observed that the THB enrichment culture (eTHB) has favored the growth of microbial species that were originally present at very low abundance in the raw freshwater fish sample (Pre), consistent with the observed increase in microbiome diversity (Pre vs eTHB: Shannon diversity increased from 0.40 to 1.02; Simpson diversity increased from 0.24 to 0.46) (Fig. 2A and B; Tables S2 and S3). Notably, these microbial species include the target pathogen species of interest, GBS, and other microbial species such as *Enterococcus faecalis* and *Lactococcus lactis* that are relevant to food safety (43) or fish health (44, 45) (Fig. 2C). Intriguingly, while BHI enrichment has been reported to enhance the possibility of the successful isolation of Streptococci (46, 47), we did not observe a substantial increase in the compositional fraction of GBS in BHI enrichment culture (eBHI) with respect to the overall fish microbiome (increase in relative abundance was less than 0.50%) when compared to the microbiome in the eTHB (Fig. 2C). Instead, we observed BHI enrichment culturing to mainly effect a reduction in the relative abundance (26.78%) of *Lactococcus* species and a corresponding increase in the relative abundance (35.85%) of *Kurthia* species (Fig. 2B). As *Lactococcus* species but not *Kurthia* species could grow in the subsequent enrichment step on the ChromID plate (Tables S2 and S3), this result suggested that eBHI enrichment culturing could be facilitating GBS isolation via the reduction of bacterial competition for GBS growth during the subsequent and final selective enrichment culture step (i.e., chromID). We also analyzed the four enrichment culture fractions (i.e., Pre, eTHB, eBHI, and chromID) with both assembly-based (i.e., *de novo*-based; Fig. S1A) and mapping-based (i.e., reference-based; Fig. 2D) analytics to recover the GBS genome from the different enrichment culture microbiomes. Among the four enrichment culture fractions, eTHB and eBHI generated the highest quality GBS genomes with almost complete (up to 95%) genome completeness (Fig. 2E; Fig. S1B).

Factoring in the requirement to concurrently maximize the detection range to include different microbial species (i.e., microbial diversity), LOD (i.e., GBS relative abundance), and TAT (i.e., time to results), the eTHB appears to be the most optimal for the application of quasimetagenomics. Leveraging on the insights generated from this pilot, we were able to establish a culture enrichment strategy optimal for the proposed metagenomics-based workflow, reducing the TAT to results by at least 50% (Fig. 1).

### Benchmarking analysis of the metagenomics-based workflow for detection and genomic characterization of GBS in raw freshwater fish

**Fig 3:**
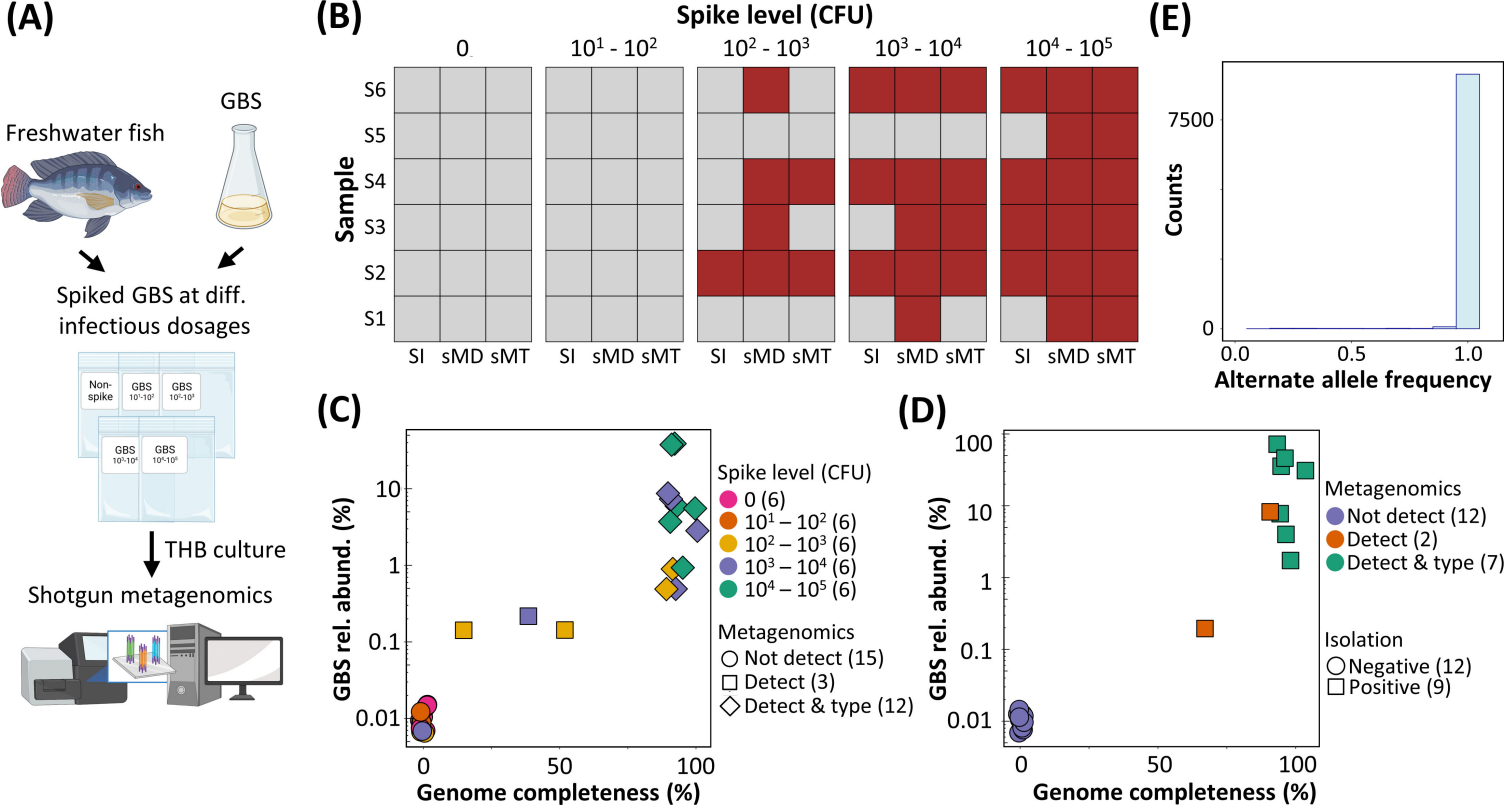
Benchmarking the limit of detection and accuracy of metagenomics-based and isolation-based workflows for the detection and characterization of GBS in raw freshwater fish samples. (**A**) Graphical illustration showing the experimental setup for one set of benchmarking sample. The figure is created with BioRender. (**B**) Heatmap summary of benchmarking results comparing the isolation-based and metagenomics-based workflow on six sets of raw freshwater fish samples that were artificially spiked with varying amounts (CFU) of GBS ST283. Brown-colored square denotes a successful detection and/or sequence typing of GBS ST283. (**C**) Metagenomics-based workflow output of the relative abundances and completeness of recovered genomes for GBS ST283 in raw freshwater fish samples artificially spiked with different amounts (CFU) of GBS ST283. Numbers in brackets denote the number of samples. (**D**) Metagenomics-based workflow output of the relative abundances and the completeness of recovered genomes for GBS in 9 real-world freshwater fish samples positive for GBS isolation (i.e., naturally contaminated) and 12 real-world freshwater fish samples negative for GBS isolation. Numbers in brackets denote the number of samples. (**E**) SNP profile of a GBS genome recovered from an eTHB fraction of raw freshwater fish sample (i.e., M0045) called against a reference GBS ST283 genome. SNPs called are predominantly at 100% frequency indicating the presence of a dominant single GBS strain. SI, isolation-based workflow; sMD, metagenomics-based workflow for GBS detection; sMT, metagenomics-based workflow for GBS sequence typing.

The metagenomics-based workflow was designed to be capable of implementing both assembly-based (i.e., *de novo*-based; Fig. S1A) and mapping-based (i.e., reference-based; Fig. 2D) analytics. Unlike mapping-based analytics, assembly-based analytics offer the advantage of not requiring a reference genome and, hence, can be used for detecting and characterizing novel (unknown) microbes. On the other hand, mapping-based analytics, through SNP frequency profiling, would be more adept (over assembly-based analytics) at elucidating the concurrent presence of multiple different microbial strains (48). Furthermore, we hypothesized that mapping-based analytics would outperform assembly-based analytics in terms of LOD as well as the completeness of the recovered targeted pathogen genome. Consequently, for the purpose of detection and genomic characterization of GBS in raw freshwater fish, since reference genomes of GBS are readily available, we defaulted (unless otherwise stated) the metagenomics-based workflow to mapping-based analytics for subsequent analysis in this study. We proceeded to benchmark our metagenomics-based workflow through conducting spike-in of GBS at varying levels into raw freshwater fish samples and then subjecting these spike-in samples to the isolation-based workflow and the metagenomics-based workflow concurrently (Fig. 3A). The metagenomics-based workflow was shown to potentially outperform the isolation-based workflow with a better LOD (for GBS) for some of the raw freshwater fish samples (Fig. 3B). Higher GBS spike-in levels were found to be associated with higher GBS relative abundance in their respective eTHB fractions and higher completeness for the GBS genomes that were correspondingly recovered (Fig. 3C). GBS genomes from the eTHB fractions of low GBS relative abundances (ranging from 0.1% to 0.2%) had low completeness (ranging from 15% to 52%) and could not be sequence-typed through the metagenomics-based workflow (Fig. 3C). Mapping-based analytics was shown to outperform assembly-based analytics in terms of LOD for GBS and completeness of recovered GBS genomes (Fig. 3B and C; Fig. S1C and D), validating our earlier hypothesis on the superiority of mapping-based analytics over assembly-based analytics for the target microbe with the available high-quality genome reference.

### Real-world performance of the metagenomics-based workflow for detection and genomic characterization of GBS in raw freshwater fish

For a more comprehensive evaluation of the real-world performance of the developed metagenomics-based workflow, we expanded our benchmarking to retrospectively apply the metagenomics-based workflow on 21 (i.e., 9 GBS-positive and 12 GBS-negative) raw freshwater fish samples that were previously tested for GBS by the isolation-based workflow (Table S4). These raw freshwater fish samples specifically included freshwater fish species (Table S4) implicated in the previous 2015 outbreak (1) (e.g., snakehead and Asian bighead carp). While a 100% concordance for GBS detection results was observed between the isolation-based and metagenomics-based workflow, the metagenomics-based workflow failed to sequence type two GBS-positive samples (Fig. 3D). As expected, unlike other GBS-positive samples that had high GBS relative abundances (>2%) and high GBS genome recovery completeness (>91% completeness) in their respective eTHB fractions, one of the failed samples was associated with a substantially lower GBS relative abundance (0.21%) and lower GBS genome completeness (65.58%) (Fig. 3D). The other GBS-positive sample had failed sequence typing due to the concurrent presence of multiple GBS strains in its eTHB fraction (Fig. S2). However, our results highlighted that the concurrent presence of multiple GBS strains in a raw freshwater fish sample is not common, with majority of tested GBS-positive eTHB enrichment cultures (seven out of eight) found to harbor only a single GBS strain (Fig. 3E; Fig. S2).

Unlike the targeted isolation-based workflow, which only tests for GBS, the metagenomics-based workflow can provide information on other microbial species in the eTHB enrichment cultures of raw freshwater fish (Tables S2 and S3). While the growth potential of GBS is known to be impacted by culture microbiome composition (49), we did not identify any unique eTHB enrichment culture microbiome signature associated with GBS status (Fig. S3A through C). Instead, the eTHB enrichment culture microbiomes were largely comprised of similar core microbial species (e.g., *Streptococcus* spp., *Lactococcus* spp., *Carnobacterium* spp., *Enterococcus* spp., and *Kurthia* spp.) independent of GBS presence (Fig. S3C).

The development of long-read metagenomics on a portable nanopore sequencing platform offered high potential for use in point-of-need testing to support rapid outbreak responses (50). While the developed short-read metagenomics-based workflow cannot be directly applied to a long-read nanopore sequencing platform, the general architecture of our short-read metagenomics workflow is sufficiently versatile for adaptation to long-read metagenomics-based workflow. To further extend the utility of our workflow to potentially support point-of-need testing, we modified the wet laboratory protocol to enable portable nanopore sequencing and develop separate long-read bioinformatics analytics for incorporation into the metagenomics-based workflow (Fig. S4A). We validated this long-read metagenomics-based workflow with a separate GBS spike-in experiment (Fig. S4B) and showed that the adapted workflow successfully detected and sequence-typed GBS in raw freshwater fish with a LOD similar to that of the isolation-based workflow (Fig. S4C).

## DISCUSSION

There are global drivers of change present in the world today that are likely to intensify foodborne outbreaks. For example, climate change can impact the biology of foodborne pathogens to increase their virulence and transmission (51–53). Increasing globalized food trades is another driver that will accelerate the transmission of foodborne pathogens worldwide (54). Consequently, foodborne pathogen outbreaks may become more prevalent, widespread, and severe. A robust, high-throughput, and rapid food microbiological testing method would be key to informing prompt and effective outbreak control measures (55). Isolation-based testing methods that can take up to weeks are clearly inadequate to support this pertinent need. Using GBS in raw freshwater fish as an example, the isolation-based workflow, which takes as long as 2 weeks to complete, typically involves three sequential culture enrichments for isolation and multiple downstream assays to identify and characterize (e.g., sequence type) the isolate (7). Furthermore, such isolation workflows are generally labor-intensive and have low throughput (56). At times, the interpretation of the phenotypic results from isolation-based workflows can be subjective. For example, the determination of positive colonies using chromogenic GBS screening media and latex agglutination tests is dependent on visual inspection, which might be ambiguous, leading to inaccurate results (57).

Metagenomics circumvents the limitations associated with the conventional isolation-based methods by enabling direct recovery of genomes without the need for isolates, thus substantially reducing the turnaround time (58). Furthermore, as the output is digital, the result interpretation can be readily automated and standardized. Analysis of the GBS genomes recovered from metagenomics-based workflow can also provide information on antimicrobial resistance genes that the GBS isolates harbored. For example, we identified *tetM* (tetracycline) and *ermB* (erythromycin) genes in some of the recovered GBS genomes from naturally contaminated fish. Another strength of the metagenomics-based workflows is their high-throughput levels, allowing for the simultaneous screening of multiple pathogens in a single assay, and they are amendable for automation (59). Remarkably, our study serves as a good illustration of the merits on the use of metagenomics for food safety testing: (i) the metagenomics-based workflow can reduce the turnaround time for GBS detection and characterization in raw freshwater fish by more than 50% (from 2 weeks to less than 5 days) compared to the isolation-based workflow, (ii) the metagenomics-based workflow’s performance in terms of LOD and accuracy to detect and characterize GBS in raw freshwater fish is comparable to, if not better than, the isolation-based workflow, (iii) by optimizing the culturing process to enrich for gram-positive microbes, the metagenomics-based workflow is able to concurrently detect and characterize multiple pathogens of food safety and fish health relevance [e.g., GBS (7), *E. faecalis* (44)*,* and *L. lactis* (45)] in raw freshwater fish, and (iv) the metagenomics-based workflow is highly versatile with three different bioinformatics analytical modes to support different applications—assembly-based mode for *de novo* detection of novel microbes with no genome reference, mapping-based mode for reference-guided microbial detection, and long-read mode to support portable nanopore sequencing for point-of-need testing.

In this work, we report the development of a metagenomics-based workflow validated for the rapid detection and characterization of GBS in raw freshwater fish. As the metagenomics-based workflow benchmarks comparably in terms of LOD to the GBS isolation-based workflow (1), this suggests that the metagenomics-based workflow can be potentially used to support outbreak investigation. The metagenomics-based work can also be applied for prospective screening to inform the prevalence of GBS. The new methodology provides a timely addition to the arsenal of testing tools to support the biosurveillance and epidemiological work critical for food safety assurance of an emerging foodborne pathogen. In addition, through detailing the process to develop and validate the metagenomics-based workflow for the detection and characterization of GBS in freshwater fish, our work highlighted the immense potential and provided an useful framework for the development of metagenomics-based tests for different foodborne pathogens in complex food matrices.

## Data Availability

Sequencing reads are available from the SRA under project PRJNA987380. The metadata, draft genome assemblies, raw data, and data analysis source codes for this study are available at https://github.com/kaehwan/metaG.

## SUPPLEMENTAL MATERIAL

Supplemental figures spectrum.03276-23-s0001.pdf Fig. S1-S4.

Table S1 spectrum.03276-23-s0002.xlsx Reference GBS genomes used in the bioinformatics pipeline.

Table S2 spectrum.03276-23-s0003.xlsx Taxonomical genus-level profiles of raw freshwater fish enrichment cultures.

Table S3 spectrum.03276-23-s0004.xlsx Taxonomical species-level profiles of raw freshwater fish enrichment cultures.

Table S4 spectrum.03276-23-s0005.xlsx Metadata of raw freshwater fish samples.

ASM does not own the copyrights to Supplemental Material that may be linked to, or accessed through, an article. The authors have granted ASM a non-exclusive, world-wide license to publish the Supplemental Material files. Please contact the corresponding author directly for reuse.
