# Supplementary material for: A metagenomics-based workflow for the detection and genomic characterization of GBS in raw freshwater fish
Source: Microbiol Spectr. 2024 May 7;12(6):e03276-23. doi: 10.1128/spectrum.03276-23 (PMC11237576; doi:10.1128/spectrum.03276-23)
Supplement: Supplemental figures — Fig. S1-S4. [file spectrum.03276-23-s0001.pdf]

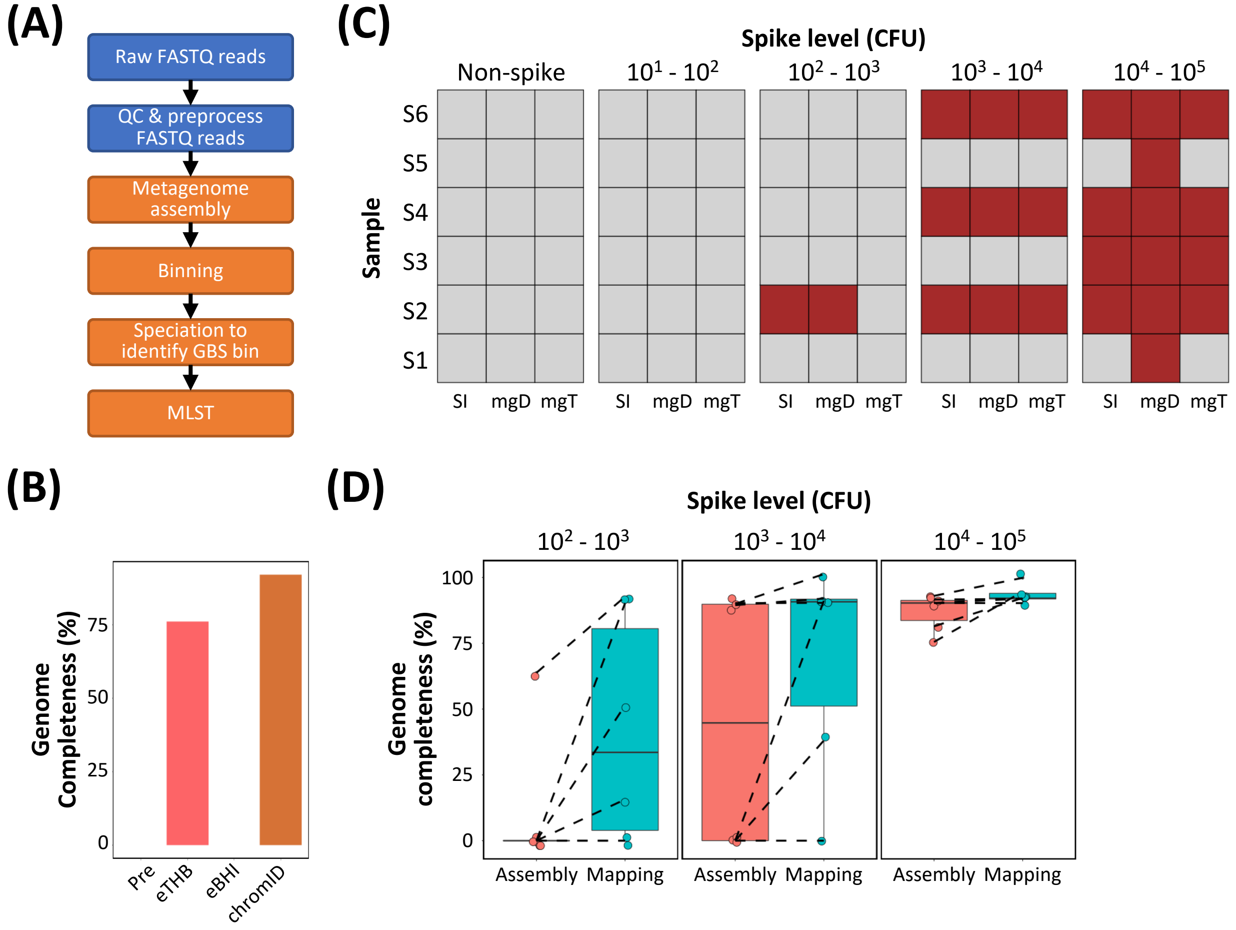

Supplementary Figure 1. Benchmarking the limit of detection and accuracy of assembly-based metagenomics-based and isolation-based workflows for the detection and characterisation of GBS in raw fresh water fish samples. (A) Outline of the assembly-based metagenomics-based bioinformatics analytics. **(B)** Completeness of recovered GBS genomes from four different culture enrichment fractions using assembly-based analytics. No GBS genome was recovered from the Pre and eBHI fractions. **(C)** Heatmap summary of benchmarking results comparing the isolation-based and assembly-based metagenomics-based workflow on six sets of raw freshwater fish samples that were artificially spiked with varying amounts (CFU) of GBS. Brown colored square denotes a successful detection and/or sequence typing of GBS. **(D)** Comparison of the recovered GBS genome completeness between the assembly-based and mapping-based analytics at various spike levels. SI: Isolation-based workflow; sMD: Assembly-based metagenomics-based workflow for GBS detection and sMT: Assembly-based metagenomics-based workflow for GBS sequence typing.

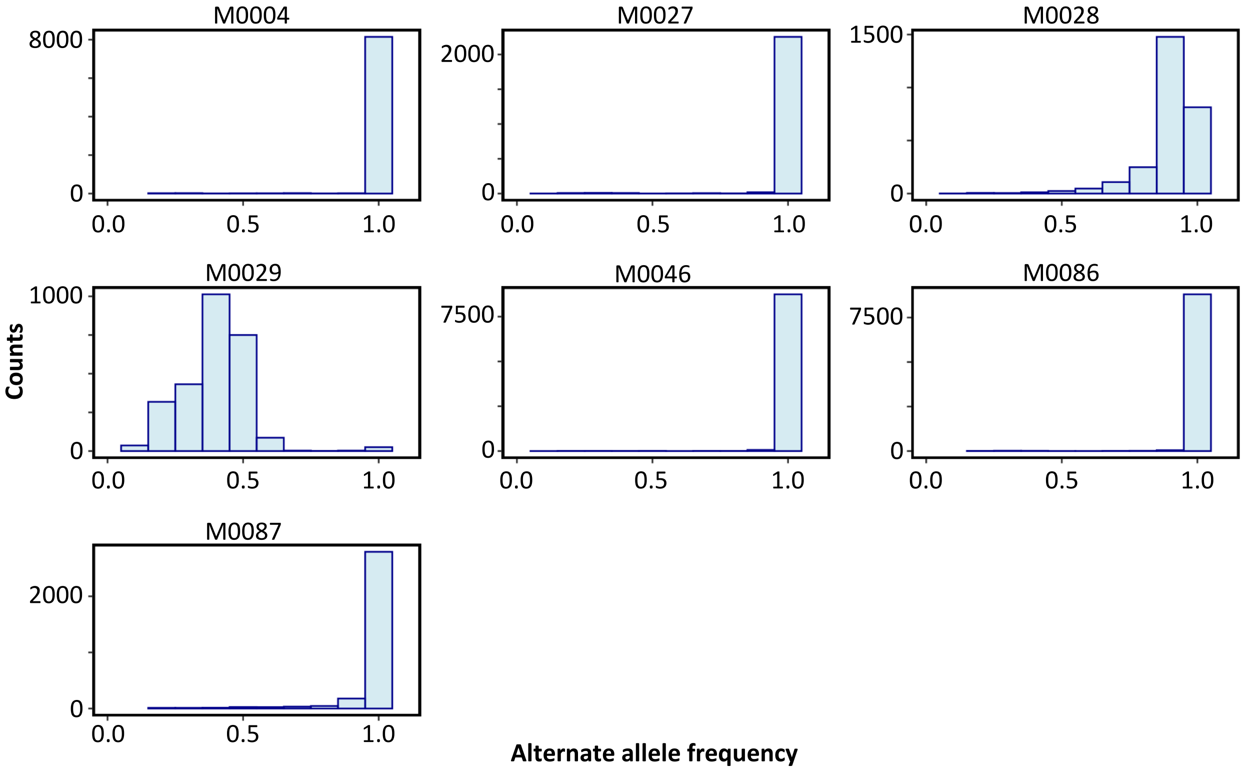

Supplementary Figure 2. Metagenomic SNP profiles of seven eTHB raw freshwater fish enrichment cultures called against a reference GBS genome. SNPs called for six of the seven cultures are predominantly at 100% frequency indicating the presence of a dominant single GBS strain. SNPs called for M0029 are mostly distributed with a range of 20-50% frequencies, suggesting the presence of multiple GBS strains.

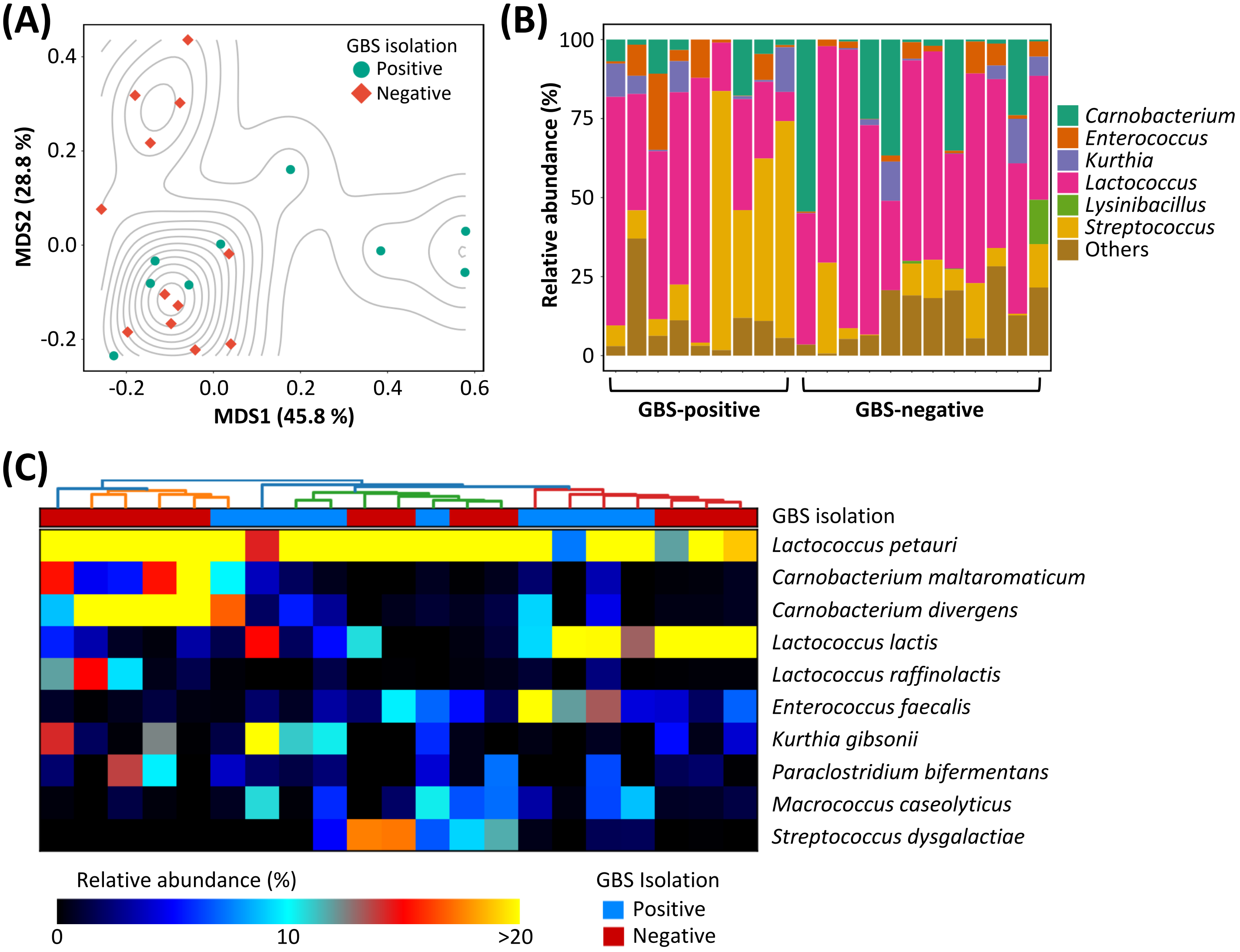

Supplementary Figure 3. No unique eTHB culture microbiome taxonomic profile signature is observed for raw freshwater fish naturally contaminated with GBS. (A) PCoA analysis of genus-level Bray-Curtis dissimilarity of raw freshwater fish eTHB microbiome taxonomic profiles. **(B)** Taxonomical genus-level profiles of the microbiomes of raw freshwater fish eTHB cultures. **(C)** Hierarchical clustering analysis of the taxonomic abundance profiles of top 10 abundant microbial species (excluding GBS) in freshwater fish eTHB culture microbiome.

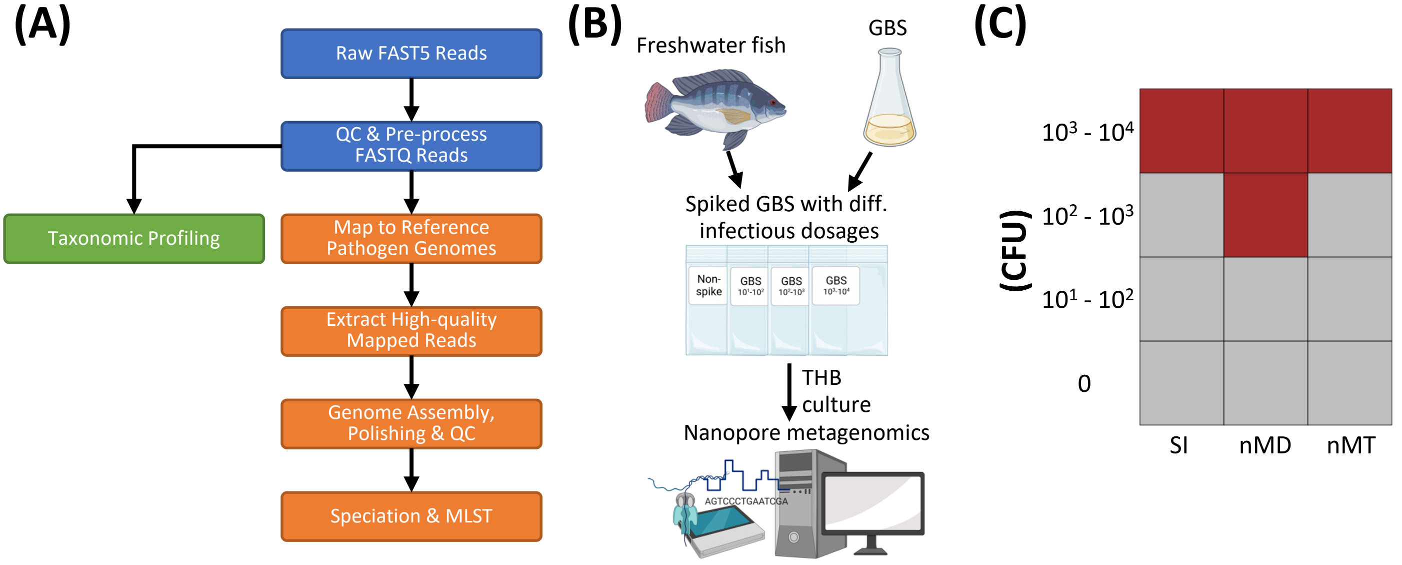

Supplementary Figure 4. Adaption of short-read metagenomics-based workflow to a long-read metagenomics-based workflow. (A) Outline of the long-read metagenomics-based bioinformatics analytics. **(B)** Graphical illustration showing the experimental setup to benchmark the long-read metagenomics-based workflow. Figure is created with BioRender (https://biorender.com). **(C)** Heatmap summary of benchmarking results comparing the isolation-based and long-read metagenomics-based workflow on one raw freshwater fish sample artificially spiked with varying amounts (CFU) of GBS. Brown colored square denotes a successful detection and/or sequence typing of GBS. SI: Isolation-based workflow; nMD: Long-read metagenomics-based workflow for GBS detection and nMT: Long-read metagenomics-based workflow for GBS sequence typing.
